# Pediatric Brain Tumors: A Clinicopathological Study and Assessment of Caregiver Quality of Life at a Tertiary Neurosurgical Referral Center in Northern India

**DOI:** 10.7759/cureus.116101

**Published:** 2026-09-11

**Authors:** Shubhi Dubey, Chhitij Srivastava, B. K Ojha, Somil Jaiswal, Sujita K Kar

**Affiliations:** 1 Department of Neurosurgery, Banaras Hindu University, Varanasi, IND; 2 Department of Neurosurgery, Apollo Hospitals, Lucknow, IND; 3 Department of Neurosurgery, King George's Medical University, Lucknow, IND; 4 Department of Psychiatry, King George's Medical University, Lucknow, IND

**Keywords:** caregiver, pediatric brain tumor, quality of life, surgical outcome, whoqol-bref

## Abstract

Background: Pediatric brain tumors are the leading cause of cancer-related death in children, yet in low- and middle-income countries (LMICs) their outcomes are shaped as much by socio-environmental factors as by tumor biology. The child and caregiver form a single therapeutic unit whose combined trajectory is rarely studied together. We analyzed the clinicopathological profile and short-term surgical outcomes of pediatric brain tumor patients alongside the quality of life (QoL) of their caregivers.

Materials and methods: In this prospective observational study, 56 consecutively admitted children (≤18 years) with histologically confirmed primary brain tumors and their principal caregivers were recruited over six months at a tertiary neurosurgical center. Clinical, operative, histopathological, and outcome data were recorded, and morbidity was graded as improved, same, or deteriorated at discharge and at three months. Caregiver QoL was measured using the World Health Organization Quality of Life-BREF (WHOQOL-BREF) questionnaire preoperatively and at three months. Categorical associations were tested with the chi-square/Fisher exact test and continuous scores with the t-test/ANOVA using SPSS Statistics version 16.0 (SPSS Inc., Chicago, IL); p < 0.05 was significant.

Results: The mean age was 10.9 ± 4.9 years, with a male predominance (60.7%, n = 34). Headache (87.5%, n = 49) and vomiting (82.1%, n = 46) were the commonest symptoms, and 53.6% (n = 30) of tumors were infratentorial. Medulloblastoma (19.6%, n = 11) was the commonest tumor, and half of all tumors were malignant. Gross- or near-total excision was achieved in 69.6% (n = 39). Three-month mortality was 30.4% (n = 17), and 58.9% (n = 33) improved overall at third-month follow-up. Benign histology, age >10 years, and full preoperative consciousness predicted successful outcome. Caregiver QoL declined significantly when the child deteriorated or died and improved when the child improved; a decline in caregiver total QoL was strongly associated with treatment failure (p < 0.001). Only 15.2% (n = 5) of children needing radiotherapy started it by three months, chiefly for financial and transport reasons.

Conclusions: Outcomes of pediatric brain tumors in this setting are governed by a web of clinical and family-level determinants. Caregiver QoL mirrors the child’s course, arguing for a holistic, family-centered model of neuro-oncological care.

## Introduction

Central nervous system (CNS) tumors are the most common solid neoplasm of childhood and, collectively, the leading cause of cancer-related death in children [1]. Population-based registries estimate an incidence of roughly two to five new cases per 100,000 children per year, with survival now exceeding 70% in well-resourced settings [1,2]. This progress, however, is unevenly distributed. In low- and middle-income countries (LMICs), where the majority of the world’s children live, survival lags far behind, a gap attributed less to differences in tumor biology than to late presentation, incomplete resection, interrupted adjuvant therapy, and treatment abandonment [3,4].

India lacks a comprehensive national pediatric brain-tumor registry, and the histological and demographic spectrum varies across its regions [5,6]. Hospital-based series remain the principal source of epidemiological insight, and reports from northern, central, and north-eastern India identify astrocytic tumors, medulloblastoma, and craniopharyngioma as the dominant histologies [6-8]. Documenting this profile at individual referral centers is essential for estimating disease load and planning the infrastructure required to manage it [7,8].

Beyond the child, the management of a pediatric brain tumor imposes a profound burden on the family. The caregiver, most often a parent, must navigate prolonged hospitalization, neurological disability, financial strain, and repeated travel to distant tertiary centers [9-11]. The World Health Organization Quality of Life-BREF (WHOQOL-BREF) instrument, which captures quality of life (QoL) across physical, psychological, social, and environmental domains, has been applied to caregiver populations in comparable settings [9,12]. Yet the child and caregiver are rarely studied as a single unit whose fortunes are intertwined.

Although Indian studies have described the clinicopathological characteristics and outcomes of pediatric brain tumors, relatively limited literature has prospectively examined the child and principal caregiver as a linked clinical unit, particularly with respect to longitudinal caregiver QoL. The relationship between the child's short-term clinical trajectory and changes in caregiver QoL is also incompletely characterized in the Indian context.

The present study therefore had two objectives. The primary objective was to describe the clinicopathological profile, surgical management, and short-term clinical outcomes of children with primary brain tumors treated at a tertiary neurosurgical referral center in northern India. The secondary objective was to assess changes in the QoL of the principal caregiver from the preoperative period to three months after surgery and to examine the association between caregiver QoL changes and the child's three-month clinical outcome.

## Materials and methods

Study design and participants

This prospective observational study, using longitudinally collected data, was conducted in the Department of Neurosurgery of King George’s Medical University, Lucknow, a tertiary care university hospital in northern India. Recruitment ran from 1st January 2017 to 30th June 2017, and each participant was followed for three months after surgery. Consecutively admitted children up to 18 years of age with radiological features of a neoplastic intracranial space-occupying lesion were screened, and those with a primary brain tumor confirmed on histopathology according to the 2007 World Health Organization (WHO) classification of CNS tumors were included, together with their principal caregiver [13]. Children over 18 years, those previously given definitive treatment for a brain tumor, and those with a known extracranial primary malignancy with intracranial metastasis or a non-neoplastic intracranial lesion were excluded. The caregiver was operationally defined as the person most involved in supporting the child, whether a biological, step, adoptive, or foster parent or legal guardian [14].

As this was a prospective observational study conducted over a predefined study period, all consecutive patients fulfilling the predefined inclusion and exclusion criteria during the recruitment period were enrolled. Hence, the sample size was determined by the number of eligible patients available during the specified period rather than by a predetermined sample size calculation. This time-bound consecutive recruitment approach was adopted to minimize selection bias and to include all eligible participants presenting during the study period. The final sample size was therefore dependent on the patient load and eligibility during the study period.

Institutional ethics committee approval and written informed consent from the caregivers were obtained.

Clinical data and outcome definition

A detailed preoperative clinical profile was recorded for every child, along with any preoperative cerebrospinal fluid (CSF) diversion, operative details, intraoperative events, postoperative complications, duration of hospital and postoperative stay, and status at discharge. Patients were reviewed at one, two, and three months; those failing to attend were traced telephonically. Histopathology and the need for adjuvant radiotherapy were documented, and at three months, the morbidity status, initiation of radiotherapy, and any re-admission or further surgery were noted. Morbidity was defined by a set of dependence indicators: level of consciousness on the Glasgow Coma Scale (GCS), presence of tracheostomy, Ryle’s tube feeding, urinary catheterization, ambulation appropriate for age, worsening or new neurological deficit, and persistence or reappearance of symptoms. On this basis, each child was classified as improved, same, or deteriorated, both at discharge and at three months relative to the preoperative baseline. Deaths during hospitalization were assigned a documented cause; probable causes were framed from caregiver interviews for deaths occurring after discharge.

Caregiver quality of life

Caregiver sociodemographic details, literacy, and socioeconomic status (modified B.G. Prasad classification) were recorded [15]. QoL was assessed using the interviewer-administered WHOQOL-BREF (Hindi version) questionnaire preoperatively (WHO-I) and at three months (WHO-II). The Hindi version was obtained from the published work of Saxena et al., who developed the Hindi WHOQOL as part of the WHOQOL project in India [16]. The instrument was accessed from the original publication and was used for the assessment of QoL in the present study, with due acknowledgement to the original authors. Formal copyright permission was subsequently obtained from the WHO for the use of the instrument in this publication [17].

Out of the 26 items in WHOQOL-BREF, the first (WHO-I Q1, WHO-II Q1) and second (WHO-I Q2, WHO-II Q2) questions recorded perceived QoL and perceived health facets, and were not included in calculating the total score. The rest of the 24 questions assessed four domains of the caregiver’s QoL. These domains and the facets in each domain are mentioned in Table 1.

**Table 1:** Domains and their facets assessed in the WHOQOL-BREF. WHOQOL-BREF: World Health Organization Quality of Life-BREF. Reproduced with permission from "WHOQOL-BREF: Introduction, Administration, Scoring and Generic Version of the Assessment: Field Trial Version, December 1996" [18].

Domain	Facets incorporated within domains
Physical health (PH)	Activities of daily living. Dependence on medicinal substances and medical aids. Energy and fatigue. Mobility. Pain and discomfort. Sleep and rest. Work capacity.
Psychological (PSY)	Positive feelings. Thinking, learning, memory, and concentration. Self-esteem. Bodily image and appearance. Negative feelings. Spirituality/religion/personal beliefs.
Social relationships (SO)	Personal relationships. Social support. Sexual activity.
Environment (ENV)	Financial resources. Freedom, physical safety, and security. Health and social care: accessibility and quality. Home environment. Opportunities for acquiring new information and skills. Participation in and opportunities for recreation/leisure activities. Physical environment (pollution/noise/traffic/climate).

Each domain had a different number of questions assigned, and a "Raw score" for each domain was calculated, which was then converted to a "Transformed score" ranging from 4 to 20 as per the method prescribed in the WHOQOL- BREF manual. A total raw score (range: 24 to 120) or a total transformed score (range: 16 to 80) was calculated by adding scores of all four domains [9,16].

Higher scores denoted better QoL throughout, and each domain score was interpreted relative to the theoretical range: a transformed domain score of 4 represents the poorest and 20 the best possible QoL in that domain, with 12 the scale midpoint. There is no universally accepted diagnostic cut-off for the WHOQOL-BREF; scores were therefore treated as continuous variables, and the principal interpretation in this study is the within-participant change between WHO-I and WHO-II, a fall indicating deterioration and a rise indicating improvement in the caregiver's perceived QoL [12,16].

Statistical analysis

Categorical variables are expressed as numbers and percentages and continuous variables as mean ± standard deviation. For comparisons between two independent groups, continuous variables were compared using the independent-samples t-test and categorical variables using the Pearson chi-square test or Fisher's exact test, as appropriate. For comparisons involving three or more groups, ANOVA was used for continuous variables. Preoperative and three-month caregiver QoL scores were compared using paired t-tests. For analysis of determinants, outcomes were dichotomized: children who improved or remained the same were classed as a successful outcome and those who deteriorated or died as a failure. Because of the modest sample size and limited number of outcome events, the analysis was restricted to univariate comparisons. Multivariable modeling was not performed because a model incorporating multiple predictors in this cohort could produce unstable estimates and risk overfitting. A p-value below 0.05 was considered significant. Analysis was performed with SPSS Statistics version 16.0 (SPSS Inc., Chicago, IL).

## Results

Clinicodemographic and radiological profile

A total of 56 children and their caregivers were studied. The mean age was 10.9 ± 4.9 years (range = six months to 18 years), with most children (59%, n = 33) aged between six and 15 years, with a male predominance (60.7%, n = 34; male-to-female ratio = 1.5:1). The mean pre-admission symptom duration was 4.0 ± 3.7 months and was significantly shorter in malignant than in benign tumors (2.7 versus 5.4 months; p = 0.006), reflecting the more aggressive course of malignant disease. Headache (87.5%, n = 49) and vomiting (82.1%, n = 46) were the commonest presenting complaints, followed by ataxia (42.9%, n = 24) and visual loss (33.9%, n = 19); most children had more than one symptom. Altered sensorium (GCS <15) was present in 17.8% (n = 10) at admission. Infratentorial tumors predominated (53.6%, n = 30), with the posterior fossa (53.6%, n = 30) and suprasellar region (23.2%, n = 13) being the commonest anatomical sites (Table 2).

**Table 2:** Clinicodemographic and radiological characteristics of pediatric brain tumor patients (N = 56). Categorical variables are presented as n (%) and continuous variables as mean ± SD. Children could report more than one presenting symptom, so symptom percentages sum to more than 100. * Others: increasing head size, irritability, altered sensorium, hearing loss, facial asymmetry, nasal regurgitation, diplopia, proptosis, and endocrinopathies. GCS: Glasgow Coma Scale; SD: standard deviation.

Characteristic	Value
Age group, years	
≤5	10 (17.9)
6-10	16 (28.6)
11-15	17 (30.4)
16-18	13 (23.2)
Age, years	10.9 ± 4.9
Sex	
Male	34 (60.7)
Female	22 (39.3)
Duration of symptoms, months	4.0 ± 3.7
Presenting symptom	
Headache	49 (87.5)
Vomiting	46 (82.1)
Ataxia	24 (42.9)
Vision loss	19 (33.9)
Others^*^	11 (19.6)
Hemiparesis	8 (14.3)
Seizure	6 (10.7)
GCS at admission	
15/15	46 (82.1)
<15 (altered sensorium)	10 (17.9)
Tumor compartment	
Infratentorial	30 (53.6)
Supratentorial	26 (46.4)
Anatomical site	
Posterior fossa	30 (53.6)
Suprasellar	13 (23.2)
Frontal lobe (right or left)	6 (10.7)
Intraventricular	5 (8.9)
Parietal lobe or optic nerve	2 (3.6)

Histopathology, operative course, and outcomes

Astrocytic tumors of various grades formed the largest histological group (30.4%, n = 17), but medulloblastoma was the single commonest tumor (19.6%, n = 11), followed by craniopharyngioma (16.1%, n = 9), pilocytic astrocytoma (14.3%, n = 8), and ependymoma (12.5%, n = 7). Benign and malignant tumors were equally represented (50% each, n = 28); craniopharyngioma was the commonest benign and medulloblastoma the commonest malignant tumor. One or the other form of (pre- or postoperative) CSF diversion was required in 62.5% (n = 35) of children. A gross- or near-total excision was achieved in 69.6% (n = 39) and a subtotal excision in 30.4% (17). The mean total hospital stay was 25.2 ± 13.0 days, and the mean postoperative stay was 10.1 ± 9.9 days. At least one postoperative complication occurred in 51.7% (n = 29) of children, most commonly a new or worsened neurological deficit (23.2%, n = 13); seven children (12.5%, n = 7) required a second operation. Overall three-month mortality was 30.4% (n = 17), of which eight deaths occurred in hospital and nine after discharge (Table 3).

**Table 3:** Histopathological spectrum, operative details, and hospital course (N = 56). Categorical variables are presented as n (%) and continuous variables as mean ± SD. Mortality figures are cumulative from the day of surgery. The denominator for initiation of radiotherapy is the 33 children in whom adjuvant radiotherapy was indicated. ** Others: oligodendroglioma, central neurocytoma, schwannoma, teratoma, and choroid plexus papilloma. CSF: cerebrospinal fluid; GTE: gross-total excision; NTE: near-total excision; STE: subtotal excision; PNET: primitive neuroectodermal tumor; ICP: intracranial pressure; SSI: surgical site infection; SD: standard deviation.

Variable	Value
Histopathology	
Astrocytic tumors (all grades)	17 (30.4)
Pilocytic astrocytoma (subset of above)	8 (14.3)
Medulloblastoma	11 (19.6)
Craniopharyngioma	9 (16.1)
Ependymoma	7 (12.5)
PNET	4 (7.1)
Epidermoid	3 (5.4)
Others^**^	5 (8.9)
Tumor behavior	
Benign	28 (50.0)
Malignant	28 (50.0)
CSF diversion (pre- or postoperative)	35 (62.5)
Extent of excision	
Gross-total or near-total (GTE/NTE)	39 (69.6)
Subtotal (STE)	17 (30.4)
Total hospital stay, days	25.2 ± 13.0
Postoperative stay, days	10.1 ± 9.9
Any postoperative complication	29 (51.8)
New or worsened neurological deficit	13 (23.2)
Endocrine or electrolyte disturbance	7 (12.5)
Postoperative hydrocephalus or raised ICP	7 (12.5)
SSI, CSF leak, or pseudomeningocele	5 (8.9)
Meningitis or ventriculitis	4 (7.1)
Re-operation within three months	7 (12.5)
Mortality	
Within 7 days	6 (10.7)
Within 1 month	7 (12.5)
Within 3 months	17 (30.4)
In-hospital deaths	8 (14.3)
Deaths after discharge	9 (16.1)
Adjuvant radiotherapy indicated	33 (58.9)
Radiotherapy started within 3 months (of 33)	5 (15.2)

At three months, 58.9% (n = 33) of children had improved relative to baseline, 1.8% (n = 1) were unchanged, 8.9% (n = 5) had deteriorated, and 30.4% (n = 17) had died. Outcomes differed significantly by histology (p = 0.047): mortality was highest for grade II diffuse astrocytoma (66.7%, n = 2 out of 3) and medulloblastoma (63.6%, n = 7 out of 11), while there were no deaths among pilocytic astrocytoma or epidermoid cases. Successful outcome was significantly more common in benign than malignant tumors (78.6%, n = 22 versus 42.9%, n = 12; p = 0.003). Adjuvant radiotherapy was indicated in 58.9% (n = 33) of children but started in only 15.2% (n = 5 out of 33) by three months; among the 84.8% (n = 28 out of 33) who could not begin it, the reasons were failure to follow up owing to financial or transport constraints (20 children) and unavailability of appointments or a non-functioning machine (eight children).

Caregiver profile and preoperative quality of life

The caregiver was the father in three-quarters of cases (75%, n = 42), the mother in 16.1% (n = 9), and an elder brother in 8.9% (n = 5); 83.9% (n = 47) were male. More than a third were illiterate (35.7%, n = 20), and a further 37.5% (n = 21) had only primary schooling. By the modified B.G. Prasad classification, 67.9% (n = 38) belonged to the lower class and 25% (n = 14) to the lower-middle class. Preoperatively, caregivers scored highest on the physical domain (mean transformed score = 15.3 ± 2.0) and social domain (15.4 ± 3.2), and lowest on the environmental domain (10.1 ± 3.0), with a mean total transformed index of 51.6 ± 11.0 (Table 4). Preoperative scores did not differ significantly between caregivers whose children subsequently improved, deteriorated, or died, indicating a comparable baseline burden across outcome groups.

**Table 4:** Caregiver profile and preoperative WHOQOL-BREF scores (N = 56). Categorical variables are presented as n (%) and WHOQOL-BREF scores as mean ± SD. Domain scores are transformed to a 4-20 scale and the total index to a 16-80 scale; higher scores indicate better quality of life. WHOQOL-BREF: World Health Organization Quality of Life-BREF; SD: standard deviation.

Variable	Value
Relation to the child	
Father	42 (75.0)
Mother	9 (16.1)
Elder brother	5 (8.9)
Caregiver sex	
Male	47 (83.9)
Female	9 (16.1)
Literacy	
Illiterate	20 (35.7)
Primary school	21 (37.5)
High school	9 (16.1)
Intermediate	4 (7.1)
Graduate	2 (3.6)
Socioeconomic class (modified B.G. Prasad)	
Lower class	38 (67.9)
Lower-middle class	14 (25.0)
Middle class	4 (7.1)
Preoperative WHOQOL-BREF, transformed score	
Physical domain (4-20)	15.3 ± 2.0
Psychological domain (4-20)	11.8 ± 2.3
Social relationships domain (4-20)	15.4 ± 3.2
Environment domain (4-20)	10.1 ± 3.0
Total index (16-80)	51.6 ± 11.0

Change in caregiver quality of life and its link to child outcome

Paired preoperative and three-month WHOQOL-BREF assessments were available for 48 of the 56 caregivers (85.7%); the eight caregivers whose children died during the index hospitalization could not complete the three-month assessment. Of these 48 caregivers, 47 belonged to one of the three outcome strata with more than one child and could be analyzed by stratum (improved, n = 33; deteriorated, n = 5; died after discharge, n = 9); the single caregiver of the one child whose status was unchanged at three months (n = 1) is not shown separately because a within-group paired comparison is not possible for a single observation, although that caregiver is included in the dichotomized analysis.

The caregiver's QoL tracked the child's clinical course closely (Table 5). Among caregivers of children who deteriorated, the total transformed index score fell from 48.4 to 38.2 (p = 0.025), with the decline reaching significance in the social relationships and environment domains. Where the child died after discharge, every domain and the total index fell sharply and significantly (total 43.3 to 22.2; p = 0.001). In contrast, caregivers of children who improved showed gains in the physical and psychological domains and in perceived QoL (p ≤ 0.005), although the modest rise in total index (53.9 to 55.2) was not significant. When outcomes were dichotomized, only one of 23 children (4.3%) whose caregiver's total QoL improved had a treatment failure, against 13 of 25 (52.0%) whose caregiver's total QoL declined (p < 0.001), establishing the change in caregiver QoL as a strong correlate of the child's outcome.

**Table 5:** Change in caregiver WHOQOL-BREF transformed scores between the preoperative assessment and the three-month follow-up, by the child's outcome at three months (n = 47 caregiver-child pairs). Paired t-test was used for all comparisons. Values are mean ± SD of transformed domain scores (range = 4-20) and total transformed index (range = 16-80); higher scores indicate better quality of life. Denominators: improved, n = 33; deteriorated, n = 5; died after discharge, n = 9. Eight caregivers of children who died during the index hospitalization did not contribute a three-month assessment, and the single caregiver of the one child whose status was unchanged is not shown because a paired comparison cannot be performed on a single observation; 33 + 5 + 9 + 1 + 8 = 56 (N). WHOQOL-BREF: World Health Organization Quality of Life-BREF; SD: standard deviation.

Outcome group	Domain	Preoperative, mean ± SD	Three months, mean ± SD	t-value	p-value
Deteriorated (n = 5)	Physical	13.8 ± 3.27	11.6 ± 4.39	2.557	0.063
	Psychological	10.6 ± 3.13	8.8 ± 3.42	1.616	0.181
	Social relationships	14.6 ± 3.51	11.4 ± 4.98	3.720	0.020
	Environment	9.4 ± 1.82	6.4 ± 2.79	5.477	0.005
	Total index	48.4 ± 9.91	38.2 ± 14.67	3.490	0.025
Improved (n = 33)	Physical	15.6 ± 1.58	16.4 ± 1.78	-3.037	0.005
	Psychological	12.2 ± 2.1	14.3 ± 2.49	-5.231	<0.001
	Social relationships	15.9 ± 2.83	15.8 ± 3.18	0.267	0.791
	Environment	10.3 ± 2.63	10.6 ± 3.44	0.089	0.930
	Total index	53.9 ± 8.07	55.2 ± 11.74	-2.548	0.016
Died after discharge (n = 9)	Physical	14.7 ± 2.78	7.0 ± 0.5	7.889	<0.001
	Psychological	11.2 ± 3.15	5.3 ± 0.87	7.005	<0.001
	Social relationships	14.4 ± 4.69	4.9 ± 0.93	6.446	<0.001
	Environment	9.7 ± 4.39	5.0 ± 1.12	5.547	0.001
	Total index	43.3 ± 18.06	22.2 ± 2.17	7.615	0.001

Determinants of child outcome

In the dichotomized analysis, 34 of 56 children (60.7%) had a successful outcome and 22 (39.3%) had a failure. A successful outcome was significantly associated with age above 10 years (21/29, 72.4% versus 13/27, 48.1% in younger children; χ² = 4.31, p = 0.038) and with benign histology (22/28, 78.6% versus 12/28, 42.9%; χ² = 7.49, p = 0.006). Children with full preoperative consciousness were more often successful than those with altered sensorium (30/46, 65.2% versus 4/10, 40.0%), but the difference did not reach significance in this small sample (Fisher exact, p = 0.167). Tumor location and extent of excision showed favorable but non-significant trends. Among caregiver factors, success was more common where the caregiver had received any schooling than where the caregiver was illiterate (24/36, 66.7% versus 10/20, 50.0%; χ² = 1.50, p = 0.221), and socioeconomic class showed no consistent gradient (χ² = 2.62, df = 2, p = 0.269), and neither reached significance. The strongest observed association was between change in caregiver QoL and child's outcome: out of 23 children whose caregiver's total QoL improved, 22 (95.7%) had a successful outcome, compared with 12 of 25 (48%) whose caregiver's QoL declined (χ² = 13.17, df = 2, p < 0.001). These findings are interpreted as univariate associations and should not be considered evidence of independent prognostic effects (Table 6).

**Table 6:** Univariate analysis of factors associated with the child's outcome at three months (N = 56). Success was defined as a child who improved or remained unchanged at three months relative to the preoperative baseline (n = 34); failure was defined as a child who deteriorated or died within three months (n = 22). Values are n (%) of the children within each factor category, so that the success and failure percentages in each row sum to 100. The Pearson chi-square test was used, except for preoperative consciousness and radiotherapy initiation, where the Fisher exact test was applied because an expected cell count was below five. All tests have one degree of freedom (df) except socioeconomic class and change in caregiver quality of life, which have two. The denominator for radiotherapy initiation is the 33 children in whom adjuvant radiotherapy was indicated, and the denominator for the change in caregiver quality of life is the 48 caregivers who completed paired assessments; all other rows use the full cohort of 56 children. GCS: Glasgow Coma Scale; GTE: gross-total excision; NTE: near-total excision; QoL: quality of life.

Factor	Category	Success, n (%)	Failure, n (%)	Test/statistic	p-value
Age	>10 years (n = 29)	21 (72.4)	8 (27.6)	χ² = 4.31	0.038
	≤10 years (n = 27)	13 (48.1)	14 (51.9)		
Preoperative GCS	15/15 (n = 46)	30 (65.2)	16 (34.8)	Fisher exact	0.167
	<15 (n = 10)	4 (40.0)	6 (60.0)		
Tumor compartment	Supratentorial (n = 26)	17 (65.4)	9 (34.6)	χ² = 0.44	0.505
	Infratentorial (n = 30)	17 (56.7)	13 (43.3)		
Extent of excision	GTE or NTE (n = 39)	24 (61.5)	15 (38.5)	χ² = 0.04	0.848
	Subtotal (n = 17)	10 (58.8)	7 (41.2)		
Tumor behavior	Benign (n = 28)	22 (78.6)	6 (21.4)	χ² = 7.49	0.006
	Malignant (n = 28)	12 (42.9)	16 (57.1)		
Radiotherapy started within 3 months	Yes (n = 5)	4 (80.0)	1 (20.0)	Fisher exact	0.625
	No (n = 28)	16 (57.1)	12 (42.9)		
Caregiver literacy	Any schooling (n = 36)	24 (66.7)	12 (33.3)	χ² = 1.50	0.221
	Illiterate (n = 20)	10 (50.0)	10 (50.0)		
Caregiver socioeconomic class	Lower class (n = 38)	25 (65.8)	13 (34.2)	χ² = 2.62	0.269
	Lower-middle class (n = 14)	6 (42.9)	8 (57.1)		
	Middle class (n = 4)	3 (75.0)	1 (25.0)		
Change in caregiver total QoL	Improved (n = 23)	22 (95.7)	1 (4.3)	χ² = 13.17	<0.001
	Declined (n = 25)	12 (48.0)	13 (52.0)		

## Discussion

This study analyzed the pediatric brain tumor patient and the caregiver as a single unit, and its findings underline that outcomes in a resource-limited setting are shaped by a web of clinical and family-level determinants. The demographic profile - a mean age of about 11 years, male predominance, and a slight excess of infratentorial tumors - is consistent with hospital-based series from India and other LMICs [6,7,19]. The dominance of astrocytic tumors as a histological group, with medulloblastoma as the single commonest tumor, mirrors both multi-institutional Indian data and international reports [2,6,8].

Headache and vomiting were the leading symptoms, as in virtually every published series of childhood CNS tumors, reflecting the frequency of raised intracranial pressure at presentation [19,20]. The significantly shorter symptom duration in malignant tumors is biologically expected and echoes observations that faster-growing, higher-grade lesions declare themselves sooner [20]. The mean pre-diagnostic interval of four months nonetheless indicates scope for earlier recognition; delays in diagnosis have repeatedly been linked to hydrocephalus, herniation, and more difficult surgery [20].

A gross- or near-total excision was achieved in roughly seven of 10 children, comparable to LMIC surgical series, and complete resection is consistently identified as a favorable prognostic factor [3,4,21]. Our three-month mortality of 30.4% is high by high-income standards but in keeping with reports from comparable settings, where early mortality is driven by advanced presentation, surgical complications, and infection rather than tumor biology alone [3,4,22]. Consistent with earlier work, benign histology, older age, and preserved preoperative consciousness predicted a successful outcome, whereas altered sensorium at presentation marked a high-risk group - findings that reproduce those of Moiyadi et al. and Menon et al. [23,24]. The trend toward better outcomes with complete resection did not reach significance within our short follow-up, as Araujo and colleagues also emphasized the primacy of resection and histology over the study interval [21].

The most striking observation of this study concerns the caregiver. Baseline WHOQOL-BREF scores were comparable across outcome groups, yet the trajectory of caregiver QoL diverged sharply with the child’s course: it fell significantly when the child deteriorated or died and rose when the child improved. The environmental and social domains were the most vulnerable, a pattern also reported among caregivers of children with brain tumors in other LMIC cohorts, where financial hardship and disrupted social participation dominate the burden [9-11]. The finding that a decline in caregiver total QoL was so strongly associated with treatment failure (p < 0.001) suggests that caregiver well-being closely tracks the patient-caregiver clinical trajectory and may serve as a useful marker of family-level treatment burden [10,25]. Whether interventions directed at caregiver well-being can improve treatment adherence or child outcomes requires prospective investigation.

Socio-environmental factors were also decisive for the delivery of care itself. Although adjuvant radiotherapy was indicated in nearly three-fifths of children, only 15.2% had begun it by three months, with financial constraints, transport difficulties, fragmented appointments, and lack of awareness as recurring obstacles. This mirrors the well-documented problem of treatment abandonment in childhood cancer across LMICs, where low socioeconomic status, low parental education, and long travel distances are the principal drivers [4,26,27]. The significant association we found between caregiver literacy and the child’s outcome, and the near-significant effect of socioeconomic status, reinforce that the family’s educational and economic resources are woven into the child’s prognosis [25,26].

These findings carry practical implications. They argue for structured, repeated caregiver counseling in accessible language; early initiation of physiotherapy and rehabilitation; active tracing of patients for follow-up; and the assignment of a medical social worker to help families navigate government financial-support schemes [10,27]. At the institutional level, forming even a small multidisciplinary working group - neurosurgery, anesthesia, radiotherapy, pediatrics, radiodiagnosis, and physiotherapy - and streamlining the registration-to-treatment pathway could reduce both delay and psychological strain. Monitoring caregiver QoL alongside the child’s clinical status may offer an early, actionable signal of a family at risk of disengaging from treatment [10,25].

The study has limitations. It was a single-center series of modest size with only three months of follow-up, so its outcome and radiotherapy findings capture a short window rather than long-term survival, and the influence of resection extent and radiotherapy may become clearer over a longer period [4,21]. Also, the smaller sample size limits generalizability to other regions and healthcare systems. Histological classification followed the WHO 2007 scheme in use at the time; contemporary integrated molecular classification would refine prognostication. The predominance of male caregivers, largely fathers, reflects local social structure and may limit the generalizability of the caregiver findings. Because of the limited sample size and number of outcome failures, only univariate analysis was performed. We did not undertake multivariable modeling, and therefore the observed associations should not be interpreted as independent prognostic effects. Nonetheless, by studying the patient and caregiver together, the study offers an integrated view that is often missing from purely clinical series.

## Conclusions

The demographic and histological profile of pediatric brain tumors at this tertiary center is consistent with published data, but their short-term outcomes are governed by a combination of clinical and family-level factors. Benign histology, older age, and preserved consciousness favor success, while the caregiver’s QoL mirrors the child’s course. In a resource-limited setting, the neurosurgeon’s role extends beyond the operation to encompass continuous appraisal of outcomes, a holistic approach to the patient-caregiver unit, and active engagement in awareness and support so that surgical effort translates into durable benefit. Future multicenter studies should use longer follow-up to determine whether improvements in family support and treatment accessibility translate into better treatment completion, QoL, and long-term outcomes.
